# Neuroprotective Effects of Purpurin Against Ischemic Damage via MAPKs, Bax, and Oxidative Stress Cascades in the Gerbil Hippocampus

**DOI:** 10.1007/s12035-021-02642-0

**Published:** 2022-01-30

**Authors:** Woosuk Kim, Hyun Jung Kwon, Hyo Young Jung, Kyu Ri Hahn, Yeo Sung Yoon, In Koo Hwang, Soo Young Choi, Dae Won Kim

**Affiliations:** 1 Department of Biomedical Sciences, Research Institute for Bioscience and Biotechnology, Hallym University, Chuncheon, 24252 South Korea; 2 Department of Anatomy, College of Veterinary Medicine, and Veterinary Science Research Institute, Konkuk University, Seoul, 05030 South Korea; 3 Department of Biochemistry and Molecular Biology, Research Institute of Oral Sciences, College of Dentistry, Gangneung-Wonju National University, Gangneung, 25457 South Korea; 4 Department of Anatomy and Cell Biology, College of Veterinary Medicine, and Research Institute for Veterinary Science, Seoul National University, Seoul, 08826 South Korea; 5 Department of Veterinary Medicine & Institute of Veterinary Science, Chungnam National University, Daejeon, South Korea

**Keywords:** Purpurin, Ischemia, Hippocampus, Microglia, Bax, Pro-inflammatory cytokine, MAPKs

## Abstract

Purpurin has various effects, including anti-inflammatory effects, and can efficiently cross the blood–brain barrier. In the present study, we investigated the effects of purpurin on oxidative stress in HT22 cells and mild brain damage in the gerbil hippocampal CA1 region induced by transient forebrain ischemia. Oxidative stress induced by H_2_O_2_ was significantly ameliorated by treatment with purpurin, based on changes in cell death, DNA fragmentation, formation of reactive oxygen species, and pro-apoptotic (Bax)/anti-apoptotic (Bcl-2) protein levels. In addition, treatment with purpurin significantly reduced the phosphorylation of c-Jun N-terminal kinase (JNK), extracellular signal-regulated kinase 1/2 (ERK), and p38 signaling in HT22 cells. Transient forebrain ischemia in gerbils led to a significant increase in locomotor activity 1 day after ischemia and significant decrease in number of surviving cells in the CA1 region 4 days after ischemia. Administration of purpurin reduced the travel distance 1 day after ischemia and abrogates the neuronal death in the hippocampal CA1 region 4 days after ischemia based on immunohistochemical and histochemical staining for NeuN and Fluoro-Jade C, respectively. Purpurin treatment significantly decreased the activation of microglia and astrocytes as well as the increases of nuclear factor kappa-light-chain-enhancer of activated B cells p65 in the hippocampal CA1 region 4 days after ischemia and ameliorated the ischemia-induced transient increases of interleukin (IL)-1β, IL-6, and tumor necrosis factor (TNF)-α in the hippocampus 6 h after ischemia. In addition, purpurin significantly alleviated the ischemia-induced phosphorylation of JNK, ERK, and p38 in the hippocampus 1 day after ischemia. Furthermore, purpurin treatment significantly mitigated the increases of Bax in the hippocampus 1 day after ischemia and the lipid peroxidation based on malondialdehyde and hydroperoxides levels 2 days after ischemia. These results suggest that purpurin can be one of the potential candidates to reduce neuronal damage and inflammatory responses after oxidative stress in HT22 cells or ischemic damage in gerbils.

## Introduction

Ischemic stroke is a life-threatening disease that affects approximately 15 million people worldwide annually [1]. Interruption of the blood flow into the brain causes a reduction in the supply of oxygen and glucose into the brain, resulting in damage to affected areas, including the hippocampus [2, 3]. Reperfusion of interrupted vessels into the brain enormously increases the blood supply to the brain, but glucose metabolism is impaired via the pyruvate dehydrogenase pathway in neurons and pyruvate carboxylase pathway in astrocytes [4]. Normally, oxygen radicals are generated from 0.2–2% of oxygen by the electron transport chain [5] and scavenged by antioxidants in the body [6, 7]. However, ischemia/reperfusion significantly increases the formation of oxygen radicals, exceeding the scavenging capacity of antioxidant enzymes in neurons, and finally causing oxidative damage and propagating inflammatory damage in neurons after ischemia [8, 9].

Many attempts have been made to prevent and reduce brain damage after ischemic damage using herbal extracts because of their high phenolic and flavonoid contents [10, 11]. Anthraquinones have a 9,10-dioxoanthracene core substituted with phenolic hydroxyl and aliphatic groups in the two benzene rings. Anthraquinones are less highlighted, although they have various biological effects that inhibit the progression of diseases [12]. Purpurin, an anthraquinone, exhibits antioxidant, anti-inflammatory, and antifungal effects in in vitro assays [13, 14] and anti-angiogenic effects in a zebrafish model [15]. In addition, purpurin inhibits monoamine oxidase and shows potential for drug development in depression [16, 17]. Purpurin is able to cross the blood–brain barrier (BBB) assessed in human brain-like endothelial cells [18], which mimic the in vivo BBB [19].

However, few studies have been conducted to elucidate the effects of purpurin against brain damage. A recent study demonstrated the neuroprotective effects of purpurin against Alzheimer’s disease-like symptoms [18], but no studies have examined the effects of purpurin against ischemic damage. Transient forebrain ischemia only causes less severe damage to brain compared to focal ischemia. Similarly, hydrogen peroxide (H_2_O_2_) induces oxidative stress and mild neuronal damage in HT22 cells compared to oxygen and glucose deprivation, which is comparable to focal ischemic models in rodents. In addition, several studies demonstrated temporal and spatial patterns of oxidative stress in the brain of gerbils after ischemia [20, 21]. In the present study, we elucidated the effects of purpurin and its mechanisms based on H_2_O_2_-induced oxidative stress in HT22 cells and ischemia-induced neuronal damage in the gerbil hippocampal CA1 region.

## Materials and Methods

### Cell preparation and Determination of Cellular Toxicity in HT22 cells

Murine hippocampal HT22 cells were obtained from ATCC (Manassas, VA, USA) and cultured in Dulbecco’s modified Eagle’s medium as described in previous studies [22, 23]. Purpurin was dissolved in 200-mM dimethyl sulfoxide (DMSO) and various concentrations of purpurin (1–200 μM) were added to HT22 cells for 60 min. The cells were then harvested to observe the cellular toxicity of purpurin in HT22 cells. Cellular toxicity was assessed by measuring the fluorescence of formazan produced using the WST-1 assay kit (Sigma, St. Louis, MO, USA) and a Fluoroskan ELISA plate reader (Labsystems Multiskan MCC/340, Helsinki, Finland) as described in previous studies [22, 23].

### Measurements of Reactive Oxygen Species, DNA Fragmentation, and Cell Viability in HT22 Cells

Cells were exposed to 25-μM purpurin or 200-mM DMSO immediately after treatment with 1-mM H_2_O_2_. For reactive oxygen species (ROS) formation, 20-μM 2′,7′-dichlorofluorescein diacetate (DCF-DA, Invitrogen Molecular Probes, Eugene, OR, USA) was added to HT22 cells at 10 min after H_2_O_2_ treatment to induce the formation of DCF, which has strong fluorescence. Cells were harvested 30 min after DCF-DA treatment. DNA fragmentation was validated using terminal deoxynucleotidyl transferase dUTP nick end labeling (TUNEL) staining as described in previous studies [22, 23]. Briefly, cells were harvested at 3 h after H_2_O_2_ treatment, and DNA fragmentation was visualized using a TUNEL staining kit (Sigma). Microphotographs from DCF-DA and TUNEL staining were taken using a confocal fluorescence microscope (LSM 510 META NLO; Zeiss GmbH, Jena, Germany), and the fluorescence intensity was measured using a Fluoroskan ELISA plate reader (Labsystems Multiskan MCC/340). Cell death was assessed using a WST-1 assay at 5 h after H_2_O_2_ treatment, and formazan fluorescence was measured using a Fluoroskan ELISA plate reader.

### Western Blot Analysis in HT22 cells

To elucidate the possible mechanisms of purpurin’s effects against oxidative stress, cells were harvested 2 h after H_2_O_2_ treatment. Thereafter, cells were lysed with ice-cold radioimmunoprecipitation assay buffer (Thermo Scientific, IL, USA), and western blotting for mitogen-activated protein kinases (MAPKs) was performed as described in a previous study [24]. Briefly, the following primary antibodies were used: rabbit anti-c-Jun N-terminal kinase (JNK), p-JNK, anti-extracellular signal-regulated kinase 1/2 (ERK), anti-p-ERK, anti-p38, and anti-p-p38, Bax, Bcl-2, and β-actin (1:2,000; Abcam, Cambridge, UK). All antibodies except β-actin were purchased from Cell Signaling (Danvers, MA, USA) and used at the same dilution (1:1000).

### Experimental Animals

Mongolian gerbils (male, 3 months old) were obtained from Japan SLC Inc. (Shizuoka, Japan), and the experimental protocols were approved by the Institutional Animal Care and Use Committee (IACUC) of Seoul National University (SNU-200313–2). Ischemic surgery was conducted as described in previous studies [22–24]. Briefly, animals were anesthetized with 2.5% isoflurane (Hana Pharm, Co., Ltd, Hwaseong, South Korea) and both common carotid arteries in neck region were exposed and they were occluded with non-traumatic vascular clip (Roboz Surgical Instrument Co., Gaithersburg, MD, USA) for 5 min. Obstruction and reperfusion of both common carotid arteries was confirmed by observing the retina artery using an ophthalmoscope (Heine Optotechnik, Herrsching, Germany). Immediately after ischemia/reperfusion, vehicle (saline containing 0.5% sodium carboxymethyl cellulose), 1, 3, or 6 mg/kg 1,2,4-trihydroxyanthraquinone (purpurin, Sigma, St. Louis, MO) was orally administered to gerbils. The dosage was chosen based on the antidepressant-like effects of 6 mg/kg purpurin treatment in mice [17]. In addition, we did not use DMSO as a vehicle for in vivo studies because it shows neuroprotective effects against ischemic damage [25].

### Spontaneous Motor Activity

Motor activity was monitored 1 day after ischemia for 60 min because hyperactivity was induced days 1 and 2 after ischemia due to functional damage in the hippocampal CA1 region [26]. In contrast, morphological damage was evident in the hippocampal CA1 region 4 days after ischemia. Traveling activity was recorded using a digital camera system (Basler 106,200, Ahrensburg, Germany), and the travel distance and duration of immobile/mobile phases were analyzed using EthoVision XT 14 (Wageningen, the Netherlands).

### Neuronal Death, Survival and Inflammatory Responses

Neuronal death and survival were assessed using histochemical and immunohistochemical staining for Fluoro-Jade C and neuronal nuclei (NeuN), respectively, as described previously [23]; Lee et al., 2020]. In addition, inflammatory responses such as glial (microglia and astrocyte) activation and nuclear factor kappa-light-chain-enhancer of activated B cells (NF-κB) expression were visualized by immunohistochemical staining for ionized calcium-binding adapter molecule 1 (Iba-1), glial fibrillary acid protein (GFAP), and NF-κB, respectively, as described previously [21, 27]. Briefly, the animals were sacrificed with a mixture of alfaxalone (Alfaxan, 75 mg/kg; Careside, Seongnam, South Korea) and xylazine (10 mg/kg; Bayer Korea, Seoul, South Korea) 4 days after ischemia and perfused transcardially with saline and 4% paraformaldehyde. Coronal serial Sects. (30-μm thickness) were made based on brain atlas between 2.0 and 2.7 mm caudal to the bregma [28]; Three to five Sects. (90 μm apart from each other) were incubated with mouse anti-NeuN antibody (1:1000; EMD Millipore, Temecula, CA, USA), rabbit anti-Iba-1 antibody (1:500; Wako, Osaka, Japan), glial fibrillary acid protein (GFAP, 1:1,000; EMD Millipore), and rabbit anti-NF-κB (1:1,100; Abcam). Sections were reacted with 3,3′-diaminobenzidine tetrachloride (Sigma) to visualize immunoreactive signals. For Fluoro-Jade C staining, the sections attached to gelatin-coated slide were sequentially incubated in 0.06% potassium permanganate for 20 min and in 0.0001% Fluoro-Jade C (Biosensis, Thebarton, SA, Australia). The number of NeuN-immunoreactive neurons and Fluoro-Jade C stained cells was calculated using OPTIMAS software (version 6.5; CyberMetrics® Corporation, Phoenix, AZ, USA). Iba-1, GFAP, and NF-κB immunoreactivities were quantified based on pixel number and gray scale using ImageJ software version 1.80 (National Institutes of Health, Bethesda, MD, USA).

### Measurements of Pro-inflammatory Cytokines

To elucidate the mechanisms of purpurin’s effects against ischemic damage, animals (*n* = 5 in each group) were euthanized with 75 mg/kg alfaxalone and 10 mg/kg xylazine 6 h and 4 days after ischemia/reperfusion, when pro-inflammatory cytokine levels were significantly increased and returned to control levels, respectively [29, 30]. In brief, interleukin (IL)-1β, IL-6, and tumor necrosis factor (TNF)-α levels were measured in the hippocampus based on comparisons with linear calibration curves generated using IL-1β, IL-6, and TNF-α standard solutions by using their respective enzyme immunoassay kits (BioSource International Inc., Camarillo, CA, USA).

### Measurements of MAPK, Bax, and Oxidative Stress in Gerbil Hippocampus

To elucidate the MAPKs and Bax pathway in gerbil hippocampus after ischemia, animals were sacrificed 24 h after ischemia. Oxidative stress induced by transient ischemia was assessed in the hippocampus 2 days after by measurements of malondialdehyde (MDA) and lipid hydroperoxides levels, which were used as indicators of lipid peroxidation to elucidate the effects of purpurin on MAPK/oxidative stress pathway. Hippocampi were obtained from the brain and homogenized. Western blotting for MAPKs, Bcl-2, and Bax was performed described above. In addition, the concentration of MDA was analyzed using the LPO-586 kit (Calbiochem, La Jolla, CA, USA) and lipid hydroperoxides were measured with ferrous oxidation-xylenol orange assay in the hippocampal homogenates described by a previous study [20].

### Statistical Analysis

All measurements were performed in order to ensure objectivity in blind conditions, by two observers for each experiment, carrying out the measures of control and experimental samples under the same conditions. Data are presented as mean with the standard deviation, and differences in means were compared and statistically analyzed using one-way or two-way analysis of variance (ANOVA) followed by Bonferroni’s post hoc test using GraphPad Prism 5.01 software (GraphPad Software, Inc., La Jolla, CA, USA).

## Results

### Neuroprotective Effects of Purpurin Against Oxidative Stress in HT2 Cells

**Fig. 1:**
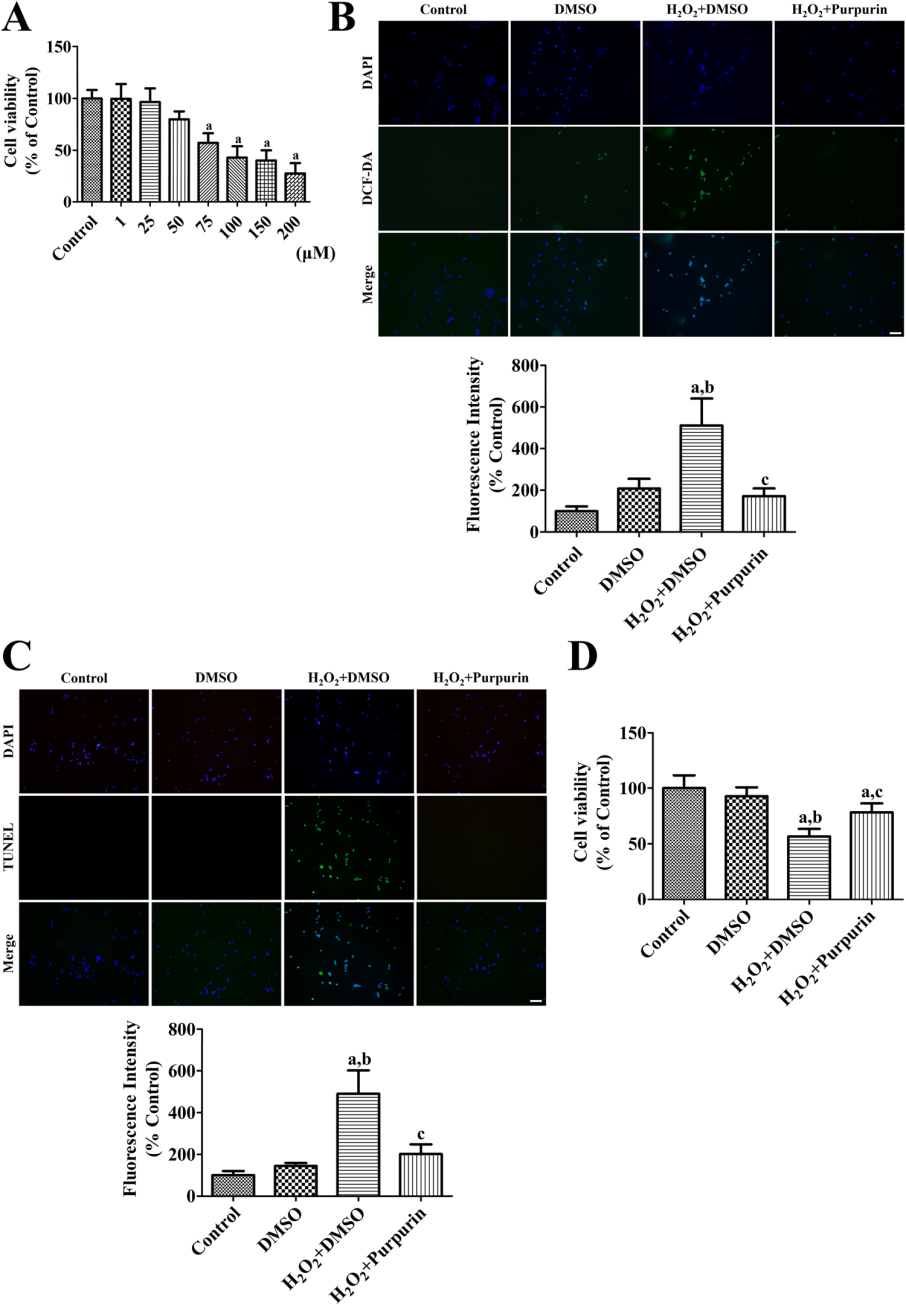
Effects of purpurin against oxidative damage in HT22 cells. **A** Concentration-dependent WST-1 assay was performed in HT22 cells to determine the optimal concentration to show minimal neurotoxicity. **B** ROS formation, **C** DNA fragmentation, and **D** cell damage was assessed after H_2_O_2_-induced oxidative stress in HT22 cells using DCF staining, TUNEL staining, and WST-1 assay. Scale bar = 50 μm. DCF and TUNEL fluorescent intensities were observed using an enzyme-linked immunosorbent assay (ELISA) reader. Data are expressed as mean value ± standard deviation and were analyzed using one-way ANOVA followed by Bonferroni’s post hoc test (^a^*p* < 0.05, significantly different from the control group; ^b^*p* < 0.05, significantly different from the DMSO group; ^c^*p* < 0.05, significantly different from the H_2_O_2_ + DMSO group)

First, we validated the toxicity of purpurin in HT22 cells to determine the effective, but non-toxic, concentration of purpurin. Purpurin treatment for 60 min showed no toxic effects at a concentration 25 μM, and higher concentrations of purpurin decreased cell viability in a concentration-dependent manner (Fig. 1A).

ROS formation was visualized by the formation of DCF fluorescence after H_2_O_2_ treatment of HT22 cells. In the control group, DCF fluorescence was faintly detected, but in the DMSO-treated group, some cells showed strong DCF fluorescence, although no statistically significant difference in DCF fluorescence was detected between the control and DMSO-treated groups. In the DMSO and H_2_O_2_-treated (H_2_O_2_ + DMSO) group, numerous DCF fluorescent cells were found, and the fluorescence intensity was significantly higher (511.3%) than that in the control group. In the purpurin and H_2_O_2_-treated (H_2_O_2_ + Purpurin) group, a few DCF fluorescent cells were found, and fluorescence intensity was significantly lower than that in the H_2_O_2_ + DMSO group (Fig. 1B).

DNA fragmentation was observed using TUNEL staining after H_2_O_2_ treatment of HT22 cells. In the control and DMSO groups, few TUNEL-positive cells were detectable among the HT22 cells and the TUNEL fluorescence intensity was low. In the H_2_O_2_ + DMSO group, many TUNEL-positive cells were observed among HT22 cells, and the fluorescence intensity was significantly increased to 490.3% of that in the control group. In the H_2_O_2_ + Purpurin group, few TUNEL-positive cells were found, and the fluorescence intensity was significantly lower than that in the H_2_O_2_ + DMSO group at 201.5% of the intensity in the control group (Fig. 1C).

Cell viability was measured using formazan fluorescence from tetrazolium salts after H_2_O_2_ treatment in HT22 cells. In the DMSO group, the cell viability was similar to that of the control group, but cell viability in the H_2_O_2_ + DMSO group was significantly lower after H_2_O_2_ treatment at 56.6% of that of the control group. In the H_2_O_2_ + Purpurin group, cell viability was significantly increased compared to that in the H_2_O_2_ + DMSO group, and cell viability in this group was at 78.3% of that of the control group (Fig. 1D).

### Neuroprotective Mechanisms of Purpurin Against Oxidative Stress in HT2 cells

**Fig. 2:**
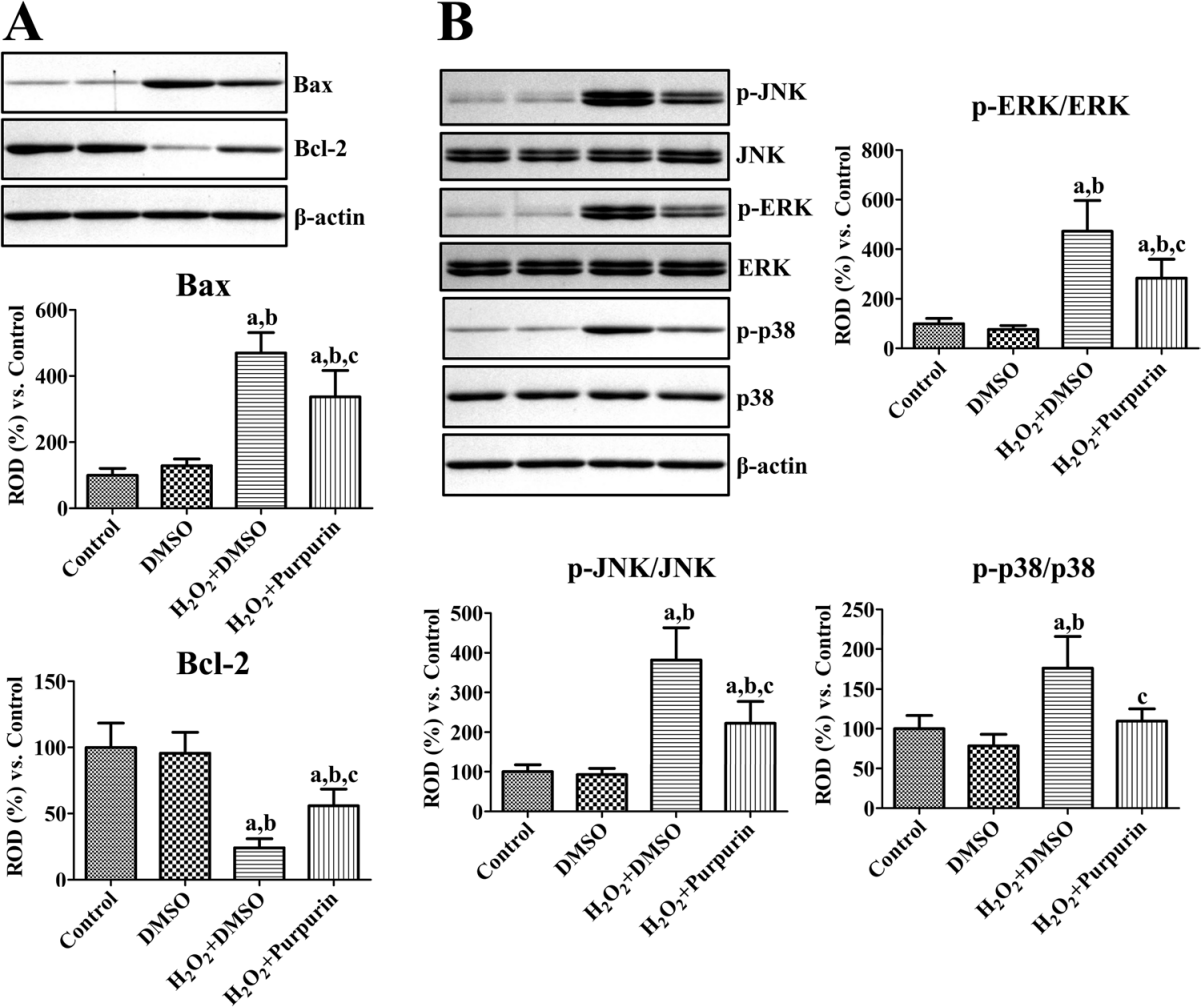
Mechanisms of purpurin’ effects against oxidative damage in HT22 cells. **A** Protein levels related to cell survival and death were measured after H_2_O_2_-induced oxidative stress in HT22 cells 2 h after H_2_O_2_ treatment using western blot analysis for Bax and Bcl-2, respectively. Protein levels of Bax and Bcl-2 were calibrated to the β-actin level. **B** Cell signaling pathway related to MAPKs were validated using western blot analysis for JNK, ERK, p38, and their phosphorylated forms 2 h after H_2_O_2_ treatment. Protein levels were converted into p-JNK/JNK, p-ERK/ERK, and p-p38/p38 ratios in each group. **A** and **B** Data are expressed as mean value ± standard deviation and were analyzed using one-way ANOVA followed by Bonferroni’s post hoc test (^a^*p* < 0.05, significantly different from the control group; ^b^*p* < 0.05, significantly different from the DMSO group; ^c^*p* < 0.05, significantly different from the H_2_O_2_ + DMSO group)

Bax and Bcl-2 protein levels were measured using western blotting after H_2_O_2_ treatment of HT22 cells. In the DMSO group, Bax and Bcl-2 protein levels did not show any significant changes relative to those in the control group. However, in the H_2_O_2_ + DMSO group, Bax protein levels were significantly higher at 469.7% of those in the control group, while Bcl-2 levels were dramatically lower at 24.2% of those in the control group. In the H_2_O_2_ + Purpurin group, changes in Bax and Bcl-2 levels were ameliorated compared to those in the H_2_O_2_ + DMSO group, respectively, and they were 336.5% and 55.9% of those in the control group, respectively (Fig. 2A).

JNK, ERK, p38 proteins, and their phosphorylated forms (p-JNK, p-ERK1/2, and p-p38) were assessed using western blotting after H_2_O_2_ treatment of HT22 cells, and the ratio of phosphorylated and naïve forms were analyzed. In the DMSO group, the p-JNK/JNK, p-ERK/ERK, and p-p38/p38 ratios were similar to those in the control group. In the H_2_O_2_ + DMSO group, the p-JNK/JNK, p-ERK/ERK, and p-p38/p38 ratios were significantly higher at 381.8%, 472.3%, and 176.4% of those in the control group, respectively. In the H_2_O_2_ + Purpurin group, the ratio of p-JNK/JNK, p-ERK/ERK, and p-p38/p38 was significantly lower than those in the H_2_O_2_ + DMSO group at 222.2%, 283.5%, and 109.7% of the ratios in the control group, respectively (Fig. 2B).

### Neuroprotective Effects of Purpurin Against Ischemic Damage in Gerbils

**Fig. 3:**
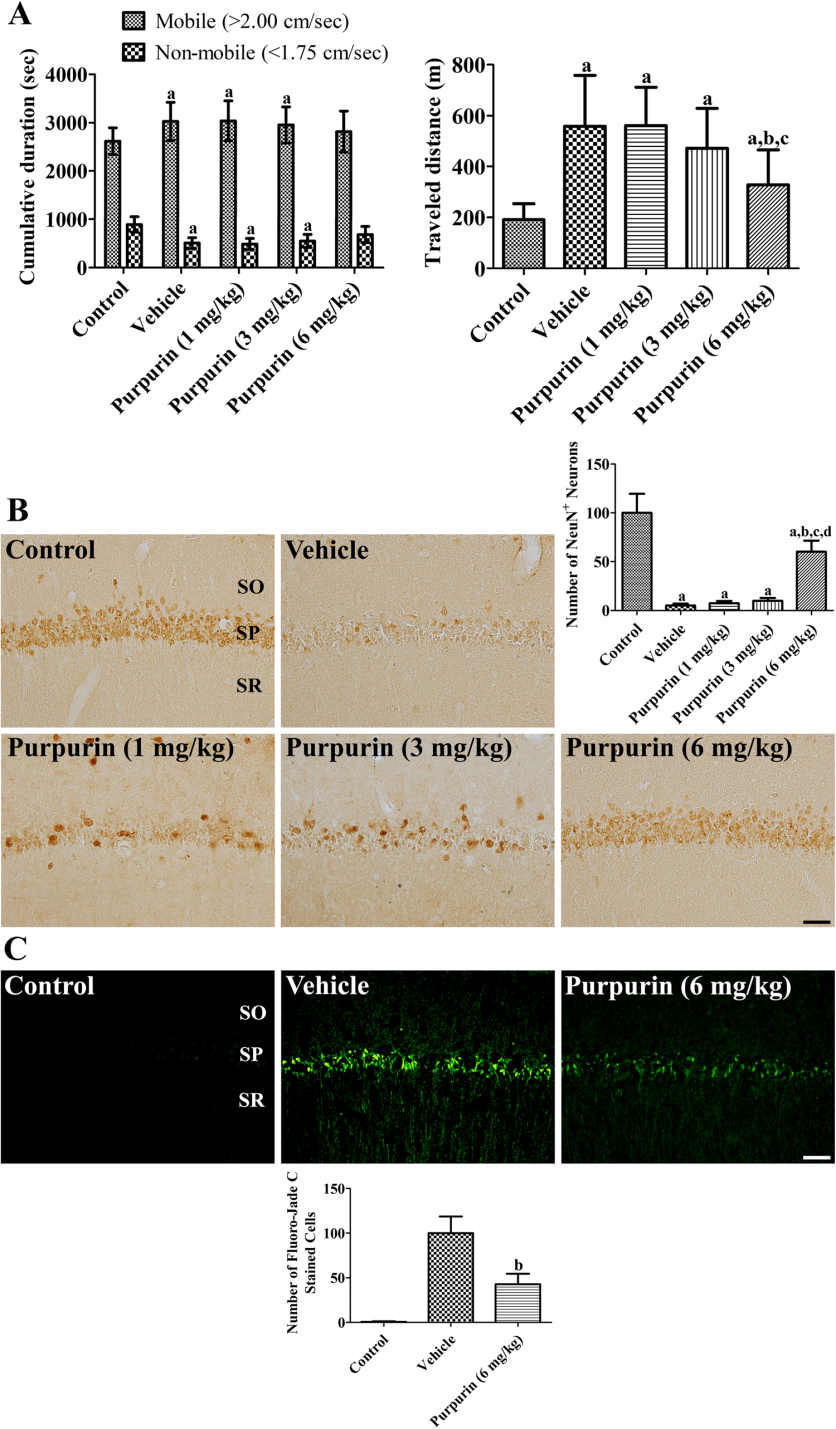
Effect of purpurin against ischemic damage in gerbils. **A** Traveled distance and cumulative duration was measured in gerbils 1 day after ischemia in sham-operated (control), ischemia-induced vehicle-treated (vehicle), and ischemia-induced purpurin-treated (purpurin) groups (*n* = 10 per group). **B** Mature neurons are visualized to show the surviving neurons after ischemic damage in the control, vehicle, and purpurin groups using NeuN immunohistochemical staining. **C** Cell death are detected in the control, vehicle, and purpurin groups using NeuN immunohistochemical staining. SO, stratum oriens; SP, stratum pyramidale; SR, stratum radiatum. Scale bar = 50 μm. The number of NeuN-immunoreactive neurons and Fluoro-Jade C stained cells is shown as a percentile value vs. control (or vehicle) group (*n* = 10 per group), respectively. **A**, **B**, and **C** Data are expressed as mean ± standard deviation and were analyzed using one-way ANOVA followed by Bonferroni’s post hoc test (^a^*p* < 0.05, significantly different from the control group; ^b^*p* < 0.05, significantly different from the vehicle group; ^c^*p* < 0.05, significantly different from the 1 mg/kg Purpurin group; ^d^*p* < 0.05, significantly different from the 3 mg/kg purpurin group)

The neuroprotective effects of purpurin were validated using locomotor behavior 1 day after ischemia. In the vehicle-treated ischemic group, the time in the mobile and immobile phases was significantly changed to 115.6% and 57.3% of those in the control group, respectively. The traveled distance in the vehicle-treated ischemic group was significantly longer than that in the control group (292.9% of that in the control group). In the 1 or 3 mg/kg purpurin-treated ischemic groups, the time spent in the mobile and non-mobile phases was similar to those in the vehicle-treated group, but in the 6 mg/kg purpurin-treated group, they did not show significant differences compared to those in the vehicle-treated group control or vehicle-treated ischemic group. Similarly, the traveled distance was significantly longer in 1 or 3 mg/kg purpurin-treated ischemic groups than in the control group. However, in the 6 mg/kg purpurin-treated group, the traveled distance was significantly less than that in the vehicle- or 1 mg/kg purpurin-treated ischemic groups (181.9% of that in the control group) (Fig. 3A).

The neuroprotective effects of purpurin were confirmed using immunohistochemical and histochemical staining for NeuN and Fluoro-Jade C in the hippocampus 4 days after ischemia. In the control group, abundant NeuN-immunoreactive cells were found in the hippocampus, while Fluoro-Jade C stained cells were very few in the hippocampal CA1 region. In the vehicle-treated ischemic group, a few NeuN-immunoreactive cells were detected in the hippocampal CA1 region (5.1% of control), whereas in other regions, NeuN-immunoreactive cells were similar levels were seen as in the control group. In contrast, Fluoro-Jade C stained cells were abundantly detected in the hippocampal CA1 region. In the 1 or 3 mg/kg purpurin-treated groups, NeuN-immunoreactive neurons were similarly observed in the hippocampal CA1 region compared to vehicle-treated group (7.5% and 9.9% of control). In the 6 mg/kg purpurin-treated ischemic group, many NeuN-immunoreactive cells were found in the CA1 region, and the number of NeuN-immunoreactive neurons was significantly higher (60.2% of control) than that in the vehicle-treated ischemic group (Fig. 3B). In this group, Fluoro-Jade C stained cells were numerous in the hippocampal CA1 region, but the number of Fluoro-Jade C stained cells was significantly decreased to 42.8% of vehicle-treated group (Fig. 3C).

### Neuroprotective Mechanisms of Purpurin’s Effects Against Ischemic Damage in Gerbils

**Fig. 4:**
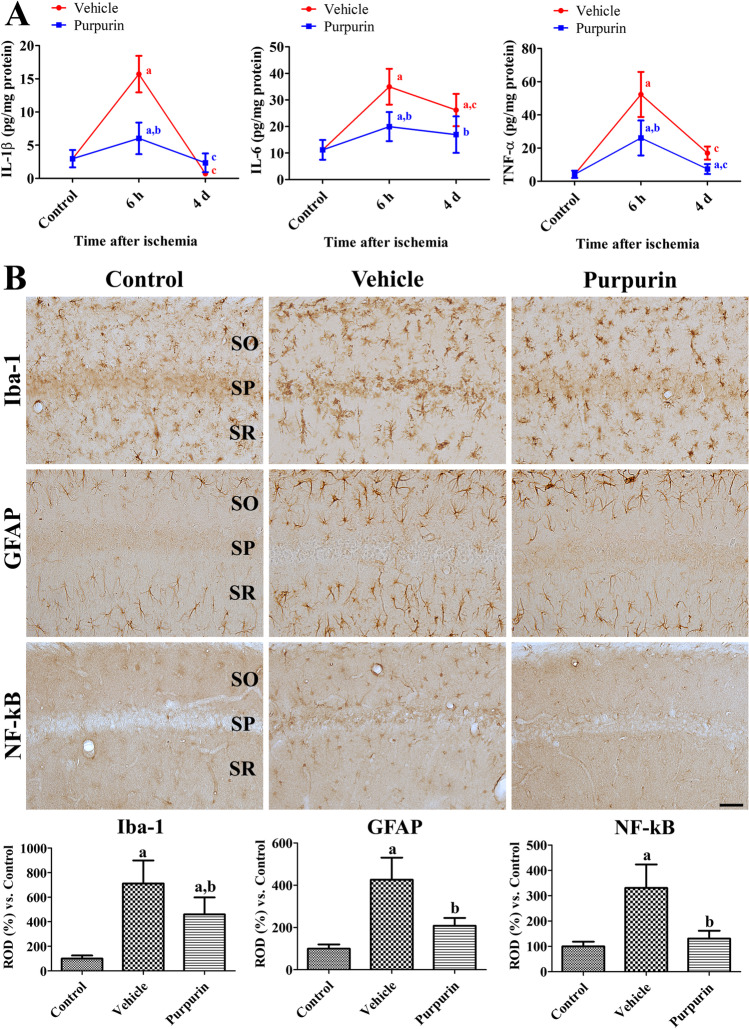
Anti-inflammatory mechanisms of purpurin against ischemic damage in gerbils. **A** Levels of pro-inflammatory cytokines were measured 6 h and 4 days after ischemia in the gerbil hippocampus of control, vehicle, and 6 mg/kg purpurin groups (*n* = 5 per group). **B** Microglia and astrocytes were visualized to show the morphological changes after ischemia in the CA1 region of the control, vehicle, and purpurin groups with Iba-1 and GFAP immunohistochemical staining, respectively. In addition, NF-κB immunohistochemical staining was conducted in the hippocampal CA1 region. SO, stratum oriens; SP, stratum pyramidale; SR, stratum radiatum. Scale bar = 50 μm. Optical density was measured and expressed as a percentage of the value vs. control group (*n* = 5 per group). Data are expressed as mean value ± standard deviation and were analyzed using one-way ANOVA followed by Bonferroni’s post hoc test (^a^*p* < 0.05, significantly different from the control group; ^b^*p* < 0.05, significantly different from the vehicle group; ^c^*p* < 0.05, significantly different from the group 6 h after ischemia)

The neuroprotective mechanisms of 6 mg/kg purpurin were evaluated in terms of anti-inflammatory responses in the hippocampus using an ELISA assay for IL-1β, IL-6, and TNF-α 6 h after ischemia. In the vehicle-treated ischemic group, IL-1β, IL-6, and TNF-α levels were significantly higher at 529.6%, 312.4%, and 1255.0% of those in the control group, respectively, 6 h after ischemia. IL-1β, IL-6, and TNF-α levels were significantly decreased 4 days in the hippocampus after ischemia compared to 6 h post-ischemic group, respectively. In the purpurin-treated ischemic group, IL-1β, IL-6, and TNF-α levels were significantly lower than those in vehicle-treated ischemic group and were 203.2%, 178.2%, and 626.1% of those in the control group 6 h after ischemia, respectively. In addition, they decreased 4 days after ischemia, and there were no significant differences on the IL-1β, IL-6, and TNF-α levels between vehicle- treated group and purpurin-treated group (Fig. 4A).

Microglia and astrocytes were visualized using immunohistochemical staining for Iba-1 and GFAP 4 days after ischemia, respectively. In the control group, Iba-1 immunoreactive microglia had a small cell body and thin processes. In addition, GFAP immunoreactive astrocytes had small cytoplasm. In the vehicle-treated ischemic group, Iba-1 immunoreactive microglia in the stratum pyramidale had a round cell body, but they had a hypertrophied cell body and thick processes in the stratum oriens and radiatum. In addition, GFAP immunoreactive astrocytes had hypertrophied and punctuated cytoplasm with thickened processes. In this group, Iba-1 and GFAP immunoreactivity was significantly increased to 711.7% and 426.3% of those in the control group, respectively. In the purpurin-treated ischemic group, Iba-1 immunoreactive microglia had a large cell body and less-developed processes compared to those in the vehicle-treated ischemic group. In addition, GFAP immunoreactive astrocytes had hypertrophied cytoplasm, but few punctuated cytoplasm. Higher in this group, Iba-1 and GFAP immunoreactivity was significantly higher than those in the vehicle-treated ischemic group and was 459.9% and 208.4% of those in the control group, respectively (Fig. 4B).

To elucidate the IL-1β- and TNF-α-mediated activation of NF-κB in the hippocampus after ischemia, NF-κB immunohistochemical staining was conducted in the hippocampus 4 days after ischemia. In the control group, weak NF-κB immunoreactivity was detectable in the hippocampal CA1 region. In the vehicle-treated group, NF-κB immunoreactive structures were detected in the stratum oriens and radiatum 4 days after ischemia and NF-κB immunoreactivity was significantly increased to 330.8% of control group. In the purpurin-treated group, NF-κB immunoreactivity was decreased to 131.0% of control group compared to that in the vehicle-treated group 4 days after ischemia (Fig. 4B).

**Fig. 5:**
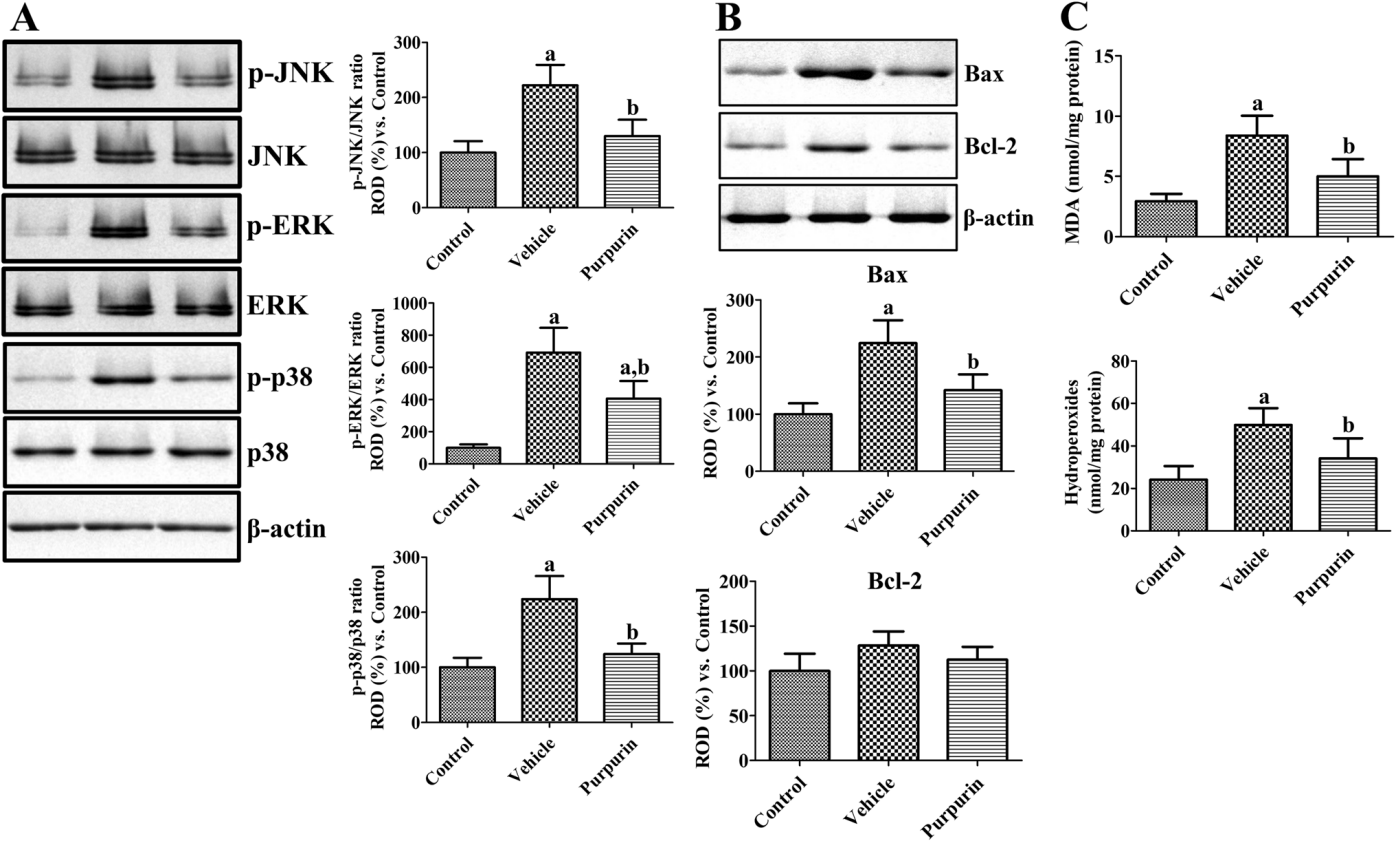
MAPKs, Bax, Bcl-2, and oxidative stress effects of purpurin against ischemic damage in gerbils. **A** Protein levels of JNK, ERK, p38, and their phosphorylated forms were validated 1 day after ischemia in the gerbil hippocampus of control, vehicle, and 6 mg/kg purpurin groups (*n* = 5 per group) using western blot analysis. Protein levels were converted into p-JNK/JNK, p-ERK/ERK, and p-p38/p38 ratios in each group. **B** Protein levels related to cell survival and death were measured 1 day after ischemia in the gerbil hippocampus of control, vehicle, and 6 mg/kg purpurin groups (*n* = 5 per group) using western blot analysis for Bax and Bcl-2, respectively. **C** MDA and hydroperoxides levels in the hippocampus 2 days after ischemia in the gerbil hippocampus of control, vehicle, and 6 mg/kg purpurin groups (*n* = 5 per group) using enzyme immunoassay. Data are expressed as mean value ± standard deviation and were analyzed using one-way ANOVA followed by Bonferroni’s post hoc test (^a^*p* < 0.05, significantly different from the control group; ^b^*p* < 0.05, significantly different from the vehicle group)

MAPKs and their phosphorylated forms were validated using western blotting 1 day after ischemia in gerbil hippocampus and the ratio of phosphorylated and naïve forms were analyzed. In the vehicle-treated ischemic group, the ratios of p-JNK/JNK, p-ERK/ERK, and p-p38/p38 were significantly increased to 221.8%, 692.4%, and 223.9% of control group, respectively, although naïve forms of MAPKs showed similar levels compared to respective control group. In the purpurin-treated ischemic group, the ratios of p-JNK/JNK, p-ERK/ERK, and p-p38/p38 were significantly lowered to 129.9%, 406.0%, and 124.2% of those in the control group compared to respective vehicle-treated ischemic group (Fig. 5A).

Bax and Bcl-2 protein levels were measured 1 day after ischemia in gerbils by western blot analysis. In the vehicle-treated group, Bax protein levels were significantly increased to 224.4% of control group. In the purpurin-treated group, Bax levels were significantly decreased compared to that in the vehicle-treated group and was 142.2% of control group. However, there were no significant differences on Bcl-2 levels among groups 24 h after ischemia (Fig. 5B).

Oxidative stress was assessed by measuring MDA and hydroperoxides levels in the hippocampus 2 days after ischemia. MDA and hydroperoxides levels were significantly increased in the vehicle-treated group to 285.1% and 206.3% of control group, respectively. In the purpurin-treated group, MDA and hydroperoxides levels were significantly decreased compared to those in vehicle-treated group and were 169.9% and 141.4% of control group, respectively (Fig. 5C).

## Discussion

Purpurin, an alizarin-type anthraquinone, has free radical scavenging activity [14, 31–33] and antioxidant effects against Trp-P-2 carcinogen by reducing DNA adducts in the liver [34]. In the present study, we investigated the role of purpurin against oxidative stress induced by H_2_O_2_ in HT22 cells and against ischemic damage in gerbils. First, we screened the toxicity of purpurin in HT22 cells to determine the optimal concentration without toxicity in HT22 cells. We observed that 25-μM purpurin was the optimal concentration with minimal toxicity in HT22 cells. The optimal concentration may differ depending on the cell type. In 3T3-L1 adipose cells, 50- and 100-μM purpurin had positive effects [33].

Oxidative stress was induced by treatment with H_2_O_2_, which increases ROS formation and decreases cell viability in a concentration-dependent manner in HT22 cells [35]. Treatment with H_2_O_2_ significantly increased ROS formation and DNA fragmentation in HT22 cells, whereas it decreased cell viability. In the present study, we analyzed the ROS formation, DNA fragmentation, and cell viability at different time points after H_2_O_2_ treatment due to ROS formation at early time points, DNA fragmentation at late period, and neuronal death at most late time point. Purpurin treatment significantly ameliorated H_2_O_2_-induced ROS formation, DNA fragmentation, and decreased cell viability in HT22 cells. This result was supported by previous studies showing that purpurin has H_2_O_2_ scavenging activity and reduces ROS levels in activated RAW 264.7 murine macrophages [14]. In addition, purpurin reduces *h*Tau accumulation in an in vitro culture system [18].

Next, we confirmed the protein levels of Bax and Bcl-2, which are the main components of the pro-apoptotic and anti-apoptotic pathways, respectively, at the same time point analyzed with cell viability, because high levels of ROS lead to mitochondrial membrane damage and release of pro-apoptotic proteins such as Bax [36]. Treatment with H_2_O_2_ significantly increased Bax levels and decreased Bcl-2 levels in HT22 cells, consistent with previous studies [35, 37]. Incubation with purpurin significantly ameliorated the changes in Bax and Bcl-2 induced by H_2_O_2_ treatment in HT22 cells. We also observed the phosphorylation of MAPKs, including JNK, ERK, and p38, because MAPKs play important roles in ROS-induced cell death and H_2_O_2_ significantly increased the expression of p-ERK 1/2, p-JNK, and p-p38 in HT22 cells [35]. Treatment with H_2_O_2_ significantly increased the p-JNK/JNK, p-ERK/ERK, and p-p38/p38 ratios in HT22 cells, and incubation with purpurin significantly mitigated the increase in the ratio.

In the present study, we also investigated the effects of purpurin against ischemic damage following oral treatment with 6 mg/kg purpurin because purpurin is able to cross the blood brain barrier [18, 38]. In addition, purpurin caused no significant changes in physiological or blood chemistry variables in an acute oral toxicity study [39]. We observed the locomotor activity 1 day after ischemia because the locomotor test is a predictive measure for assessing neuronal damage in the hippocampus during the first 2 days after ischemia and thereafter the locomotor activity is decreased gradually [40, 41]. Transient forebrain ischemia significantly increased the travel distance and time in the mobile phase, indicating hyperactivity in gerbils 1 day after ischemia. Purpurin treatment significantly reduced the travel distance and time in the mobile phase, which suggests the reduction of functional damage in the hippocampus 1 day after ischemia. In addition, we observed that 6 mg/kg, not 1 or 3 mg/kg, purpurin treatment ameliorated the ischemia-induced reduction in NeuN-immunoreactive neurons in the hippocampal CA1 region. We confirmed that treatment with 6 mg/kg purpurin significantly decreased ischemia-induced degenerating neurons in the hippocampal CA1 region based on the Fluoro-Jade C staining, which is a reliable marker for degenerating neuronal cells during all differentiation stages [42]. This result suggests that purpurin has the potential to reduce neuronal death induced by ischemia.

To elucidate the possible role of 6 mg/kg purpurin against ischemia, we observed the morphology of astrocytes and microglia as well as pro-inflammatory cytokines in the hippocampus because a recent study showed the anti-inflammatory roles of purpurin in RAW 264.7 murine macrophage cells [14]. The animals were sacrificed 6 h after ischemia to measure IL-1β, IL-6, and TNF-α levels in the hippocampus because these levels are significantly increased in the early period of ischemia [29, 30, 43]. In addition, the IL-1 receptor antagonist showed neuroprotective effects against ischemic damage in rats [44]. In the vehicle-treated group, IL-1β, IL-6, and TNF-α levels were significantly increased 6 h after ischemia/reperfusion compared to those in the control group. In the purpurin-treated ischemic group, IL-1β, IL-6, and TNF-α levels were dramatically lower in the hippocampal homogenates. This result suggests that purpurin treatment significantly reduces the release of pro-inflammatory cytokines in the hippocampus 6 h after ischemia. However, in the present study, we did not observe any significant differences on the IL-1β, IL-6, and TNF-α levels between vehicle- and purpurin-treated group 4 days after ischemia/reperfusion.

Next, we confirmed the activation of microglia and astrocytes based on their morphologies in the hippocampal CA1 region 4 days after ischemia. In the vehicle-treated group, Iba-1 immunoreactive microglia had hypertrophied cell body and thickened processes (activated microglia), and the phagocytic form (round cell body without processes) of microglia was also found in the stratum pyramidale of the CA1 region 4 days after ischemia/reperfusion. In addition, GFAP immunoreactive astrocytes had hypertrophied and punctuated cytoplasm (reactive astrocytes) in the hippocampal CA1 region, which has functions as phagocytes after transient ischemia injury [45]. This result was consistent with previous studies showing that ischemia-induced activation of microglia and astrocytes and their morphological changes in the hippocampus [46–49]. Treatment with purpurin reduced the phagocytic form of microglia in the stratum pyramidale and reactive astrocytes in the stratum radiatum and oriens. Overall Iba-1 and GFAP immunoreactivity was significantly decreased in the hippocampal CA1 region compared to those in the vehicle-treated group. A molecular docking study demonstrated that purpurin had a strong inhibitory effect on the nucleotide-binding domain leucine-rich repeat and pyrin domain containing receptor 3, which is one of the main contributors to neuroinflammation [50]. NF-κB is one of the important mediators in pathological process of ischemic damage, which can be activated by several inflammatory mediators [51]. In addition, NF-κB was expressed in the astrocytes 2 days after ischemia in gerbils and peaked 4 days after ischemia [52]. In the present study, we observed significant increases of NF-κB immunoreactivity in the hippocampus 4 days after ischemia and the treatment with purpurin significantly decreased in the hippocampal CA1 region.

In the present study, we also observed that the ischemia significantly increased the ratios of p-JNK/JNK, p-ERK/ERK, and p-p38/p38 in the gerbil hippocampus 1 day after ischemia result. This result is consistent with in vitro study in HT22 cells that oxidative stress induced by H_2_O_2_ treatment significantly increased the phosphorylation of MAPKs. In addition, several studies demonstrate the increases of MAPK phosphorylation in the hippocampus after ischemia [24, 53, 54] and treatment with JNK blocker ameliorates the neuronal death induced by ischemia [55]. In addition, the close relationship has been reported between the cytokine-related inflammation and MAPKs [56, 57]. In the present study, we observed that the purpurin treatment significantly decreased the activation of MAPK pathway in the hippocampus after ischemia.

We observed Bax and Bcl-2 levels in the hippocampus 24 h after ischemia. In addition, we measured MDA and hydroperoxides levels in the hippocampus 2 days after ischemia to elucidate the mechanisms of MAPKs, Bax, and oxidative stress cascades activation. Transient forebrain ischemia significantly elevated Bax levels 1 day after ischemia, while Bcl-2 protein levels did not show any significant difference between groups although slight increases of Bcl-2 protein was found in vehicle-treated ischemic group. This result was supported by previous studies that transient forebrain ischemia increases both apoptotic and anti-apoptotic pathways after ischemia, but the increase of anti-apoptotic signals was relatively low [58, 59]. Treatment with purpurin significantly ameliorated the increases of Bax levels induced by ischemia, suggesting that treatment with purpurin decreases ischemia-induced apoptotic pathway. Oxidative stress measured by MDA and hydroperoxides levels was significantly increased 2 days after ischemia to confirm the antioxidant effects of purpurin in the hippocampus after ischemia. Treatment with purpurin significantly abrogated the ischemia-induced oxidative damage in the hippocampus. This result suggests that purpurin has potentials to reduce the ischemia-induced oxidative damage such as MDA and hydroxides.

## Conclusions

The current findings suggest that purpurin may be a strong neuroprotective agent in HT22 cells and gerbil hippocampus to ameliorate MAPKs, Bax, and oxidative stress cascades activation after oxidative stress or ischemic damage.

## Data Availability

The datasets and supporting materials generated during and/or analyzed during the current study are available from the corresponding author on reasonable request.
